# Enabling Fairness in Healthcare Through Machine Learning

**DOI:** 10.1007/s10676-022-09658-7

**Published:** 2022-08-31

**Authors:** Thomas Grote, Geoff Keeling

**Affiliations:** 1 Ethics and Philosophy Lab; Cluster of Excellence: Machine Learning: New Perspectives for Science, University of Tübingen, Maria von Linden Str. 6, D-72076 Tübingen, Germany; 2 Institute for Human-Centered AI and McCoy Family Center for Ethics in Society, Stanford University, 450 Serra Mall, 94305 Stanford, CA USA

**Keywords:** Fairness, Machine learning, Healthcare, Bias, Decision-making

## Abstract

The use of machine learning systems for decision-support in healthcare may exacerbate health inequalities. However, recent work suggests that algorithms trained on sufficiently diverse datasets could in principle combat health inequalities. One concern about these algorithms is that their performance for patients in traditionally disadvantaged groups exceeds their performance for patients in traditionally advantaged groups. This renders the algorithmic decisions unfair relative to the standard fairness metrics in machine learning. In this paper, we defend the permissible use of affirmative algorithms; that is, algorithms trained on diverse datasets that perform better for traditionally disadvantaged groups. Whilst such algorithmic decisions may be unfair, the fairness of algorithmic decisions is not the appropriate locus of moral evaluation. What matters is the fairness of final decisions, such as diagnoses, resulting from collaboration between clinicians and algorithms. We argue that affirmative algorithms can permissibly be deployed provided the resultant final decisions are fair.

## Introduction

There are growing concerns that the proliferation of machine learning (ML) algorithms in healthcare reinforces health disparities. It is well-established that ML algorithms used for image-based medical diagnosis, risk prediction, and informing triage decisions underperform for disadvantaged groups, such as women, or racial and ethnic minorities (Adamson & Smith, 2018; Noor, 2020; Owens & Walker, 2020).^1^ However, there is also a different side to the story, as researchers in health economics and epidemiology increasingly leverage ML algorithms with the aim of identifying and even addressing health inequalities (Obermeyer et al., 2019; Chang et al., 2021). This paper articulates an account about how to manage the promise and perils of fair ML in health contexts by drawing on the insight that these systems are best seen as collaborative tools rather than straight decision-making tools.

While others have discussed this possibility, the collaboration between humans and algorithms has mostly been understood as a form of epistemic peer (dis)agreement (Bjerring and Busch, 2020; Grote & Berens 2020; Grote & Berens, 2021). Contrary to such a view, we argue that utilizing ML algorithms for the purposes of *mitigating* health disparities requires us to explore alternative models of collaboration. For this purpose, we discuss a study by Pierson et al., (2021), which developed a ML algorithm that measures pain severity for knee osteoarthritis based on X-ray images – while being trained with a racially and socioeconomically diverse dataset. A potential caveat of this diverse dataset is that the algorithm might be more sensitive to pain-relevant statistical associations for disadvantaged sub-populations than for advantaged sub-populations. Consequently, the algorithm would perform worse for advantaged sub-populations than for disadvantaged ones. Against this backdrop, our main argument is that alternative forms of collaboration between ML algorithms and clinicians can promote fairness in healthcare, even if the ML algorithm is biased.

Here, the distinction between *(un)fair algorithmic decisions* and *fair final decisions* – is a key concern (Green & Chen, 2019; Hedden, 2021; Grote and Keeling, 2022). Roughly, an algorithmic decision is a regression score or classification prediction outputted by an algorithmic, and a final decision is, for example, the diagnosis or treatment recommendation made by the clinician. The fairness debate in ML tends to silo algorithms by focussing merely on statistical properties indicative of algorithmic biases (Fazelpour et al., 2021a). This framing ignores that in practice the envisioned role of ML algorithms in clinical environments is not to decide in isolation, but to guide clinicians’ decisions. To underscore the relevance of this distinction studies within the context of criminal justice indicate that humans are susceptible to racial bias when evaluating algorithmic risk assessments: for the same risk score, Black defendants were judged more harshly than White defendants (Green & Chen, 2019). In carefully theorizing whether and under what conditions biased algorithms can permissibly be deployed in clinical settings for the purpose of mitigating health disparities, we hope to lay the grounds for an outward-looking moral evaluation of ML algorithms, as opposed to an inward-looking, focussing merely on an algorithm`s statistical properties – in which the collaboration of clinicians and ML algorithms is a cornerstone. A secondary aim is to develop a pronounced view of the difficulties in remedying health inequalities through technology, by highlighting the ethical caveats that alternative models of clinician-ML-collaboration entail.

The paper is structured as follows: Sect. 2 introduces the ML algorithm, developed by Pierson et al., (2021), as a case study. Also discussed will be different sources of bias in measurement of pain for socially and racially disadvantaged groups. Section 3 gives a brief overview on metrics of algorithmic fairness, while emphasizing the need to move beyond the statistical properties of ML algorithms. Section 4 develops a taxonomy of different types of clinician-ML-collaboration. On this basis, Sect. 5 examines how in some of these types, the interplay of an algorithm overfitting for socially and racially disadvantaged groups and a clinician might result into fair decisions. Finally, in Sect. 6, we address a potential critique of our approach, namely the charge of ‘solutionism’. This also enables us to discuss the complexities in using ML-based solution for overcoming health inequalities at a broader level.

## **2**. **Case Study: Mitigating Bias in Pain Diagnosis**

At least since the publication of ProPublica`s assessment of the COMPAS algorithm (Angwin et al., 2016) – used by many states in the US to inform pre-trial decisions and the study on skin-type bias by Buolamwini and Gebru (2018), there have been growing concerns that the deployment of ML algorithms used to make/inform consequential decisions reinforces structural inequalities. In turn, philosophers, computer scientists, and researchers from cognate fields are increasingly paying attention to the *mechanisms* through which ML algorithms disadvantage certain social groups (Barocas et al., 2019; Fazelpour & Danks, 2021; Zimmermann & Lee-Stronach, 2021).

A more recent phenomenon, by contrast, is to use ML algorithms specifically for the purpose of detecting unfair treatment. Consider some examples: a study examining a ML algorithm used to optimize the allocation of health resources found that traditional metrics of prediction quality – such as health costs – may put Black patients at a disadvantage, (Obermeyer et al., 2019, p. 453). Likewise, in analysing human mobility patterns derived from cell phone data and integrated into an SEIR-model, researchers captured mechanisms for higher Covid-19 infection risks among disadvantaged social and racial groups (Chang et al., 2021). The novelty in the study by Pierson et al., (2021) is that they developed a ML algorithm precisely for the purpose of *remedying* health disparities (see also Rajkomar et al., 2018).^2^

Their emphasis lies on the undertreatment of pain for Black people, proving to be a persistent issue in healthcare for decades (Green et al., 2003; Anderson et al., 2009): Given the same signs and symptoms, Black people are likely to be given either no medication or weaker medication than White patients. The undertreatment of pain has severe quality-of-life and socioeconomic implications for them. Causes for undertreatment are typically attributed to a combination of socioeconomic factors (e.g., Black patients may have less access to treatment) and racial stereotypes among medical professionals. As an example, a study by Hoffmann et al. (2016) found that clinicians falsely assume Black people to be less sensitive to pain. This is due to incorrect beliefs with respect to Black people having a thicker skin, less sensitive nerve endings, and being more tolerable to heat.

A recent study by Pierson et al., (2021) provides another explanation for pain disparities, by identifying biases in standard pain metrics, such as the Kellgren-Lawrence grade (KLG) – being used to classify radiographic osteoarthritis when judging the pain severity from X-ray images. Here, people of lower income or of education, or members of disadvantaged racial sub-populations experience higher pain as their more privileged counterparts in the US. Importantly, the disparities only decrease slightly after controlling for the severity of the disease by objective radiographic measures.

It is unclear what the sources of the relevant pain disparities are: they might either be rooted in factors internal to the knee or in psychological (higher stress) or social (e.g., physically more demanding jobs) factors. Pierson et al. assume that the pain disparities are rooted in knee-internal factors – without, however, providing a causal explanation for their hypothesis.

An underlying problem with KLG is that it was developed in the 1960`s within a White British population – leading a different lifestyle and arguably having different physiology than current US American populations. For this reason, the researchers assume that KLG is unable to account for some physical causes of pain in Black people or other socially disadvantaged groups (p. 136). To remedy disparities in pain diagnosis, the researchers trained a deep learning-based ML algorithm to predict pain severity from X-ray images of knees – with the aim of generating a more accurate pain metric. The training data was taken from a racially and socioeconomically diverse sample of patents in the United states (with 20% of the patients being Black, while also having many lower-income patients). Importantly, the patients also reported a knee-specific pain score (KOOS)^3^, derived from a multi-item survey on pain experienced during various activities, such as stretching the knee. Due to its holistic nature, KOOS is supposed to constitute the ‘ground truth’ – an external standard, measuring disparities in pain severity grading for KLG and the algorithm.

While KLG could only account for a small fraction in the pain disparities for disadvantaged social and racial groups, the algorithm fared much better in this respect. As there is a close link between diagnosed pain severity in KLG and the prescription of surgery, the algorithmic metric might increase the eligibility for surgery among Black patients – rather than being prescribed opioids.^4^ That being said, the researchers acknowledge some limitations of their study: the opacity of the (deep learning) algorithm makes it difficult to interpret which features in the knee are used as predictors and the algorithm might be biased – even though this time, the bias might affect traditionally privileged populations, such as white men (p. 139).^5^ While it needs to be emphasized that the worry concerning algorithmic bias is speculative, we assume for the remainder of the paper, that the algorithm`s accuracy is indeed higher for Black patients than it is for white patients.^6^

Whilst it might be preferable to deploy algorithms in healthcare, generalising equally well for all groups of patients, the deployment of algorithms tailored towards certain sub-populations may have its benefits. Afterall, one might argue that this is precisely what the project of personalized medicine is about. However, it is also clear that such an approach entails various caveats. If various algorithms for different sub-populations are being used, clinicians will have to juggle with competing diagnostic standards. For them, this might represent an excessive demand, which in turn leads to misdiagnoses. Hence the need to be exceedingly wary of how given ML algorithms are being used in clinical environments. Finally, since it is very unlikely that every algorithm will perform equally well for their given sub-population, there are serious considerations of fairness which need to be addressed. To underpin the relevant moral dialectic, we now turn to statistical fairness criteria in ML.

## On Statistical Fairness Criteria in Machine Learning

Disputes over fairness in ML typically focus on operational definitions of fairness based on statistical features of algorithmic decisions.^7^ These statistical fairness criteria compare the predictive performance of the ML algorithm between a disadvantaged and advantaged social group. If for them, the predictive performance is equal, this is seen as evidence for the fairness of the algorithm. In that respect, it is important to emphasize that – despite carrying normative baggage – statistical fairness criteria are not to be conflated with a full-fledged normative theory of fairness in algorithmic decision-making.^8^ However, since our emphasis lies on fairness in relation to diagnostic accuracy for different groups, statistical fairness criteria capture the normative key concerns. In what follows, we will briefly discuss some of the basics of statistical fairness criteria, whilst arguing for the need to switch the locus of moral evaluation from the algorithm`s statistical properties to the final decision made by a clinician.

To look at some of the operationalisations of statistical fairness criteria, consider a simple binary classification problem (such as medical diagnosis) in which a model predicts whether patients are ‘positive’ or ‘negative’ for some condition. We use Black and white patients as representative groups:

### Equal false positive rates

The fraction of Black patients who are in fact negative but are predicted positive relative to the Black population is equal to the fraction of white patients who are in fact negative put are predicted positive relative to the white population.

### Equal false negative rates

The fraction of Black patients who are in fact positive but are predicted negative relative to the Black population is equal to the fraction of white patients who are in fact positive but are predicted negative relative to the white population.

### Predictive parity

The fraction of Black individuals who are predicted positive who are in fact positive relative to the Black population is equal to the fraction of white patients who are predicted positive and are in fact positive relative to the white population.

The statistical fairness metrics characterised here are provably inconsistent except under very specific conditions that are in practice unattainable (Kleinberg et al., 2016; Chouldechova, 2017; Miconi, 2017).

To illustrate: Suppose that for Black and white patients the prevalence of the condition differs between their respective racial groups. In particular, suppose that the base rate for the condition is higher in the white population than in the Black population. What this means is that the fraction of white patients who in fact have the condition relative to the white population is greater than the fraction of Black patients who in fact have the condition relative to the Black population. Then suppose that the model satisfies predictive parity for Black and white patients. The problem is that under these conditions, either the algorithm’s predictions are perfect or the false positive and false negative rates differ for Black and white patients. This is a straightforward consequence of the differing base rates across both populations. Because equal base rates rarely obtain and because perfectly accurate classifiers are unattainable, the competing statistical definitions of what fairness consists in cannot jointly be satisfied in practice. Consequently, when selecting an appropriate statistical fairness metric, ML developers will need to balance different trade-offs – such as whether it is more important to focus on false positive or false negative diagnoses in clinical environments Biddle, 2020.

Moreover, there are various caveats to consider when implementing statistical fairness criteria into (clinical) practice. For example, Alex Beutel et al. (2019) argue that while ‘equal false positive rates’ provides philosophical guidance in that it emphasizes the importance of ensuring equality of opportunity, it is oftentimes unclear how the metric ought to be calculated. In turn, they suggest ‘conditional equality of opportunity’ as a novel fairness metric, better able to account for varying base rates across different groups. Finally, Rajkomar et al., (2018) provide an useful overview about how abstract egalitarian ideals can be technically implemented – with a view on equal patient outcomes, performance, and resource allocation.

### Algorithmic Fairness and Final Fair Decisions

Statistical fairness metrics at best capture part of what fair algorithmic decision-making amounts to in the context of healthcare, when the focus lies solely on the statistical properties of the algorithm. This is because in almost all cases machine learning systems used in healthcare are ‘decision support tools’. What this means is that the role of the system is to aid a healthcare professional in making a certain class of decisions. For example, diagnostic decisions or treatment recommendations. Accordingly, the Green and Chen (2019) and Hedden (2021) distinguish between ‘algorithmic decisions’ and ‘final decisions’. Here, algorithmic decisions are the decisions made by an algorithm, e.g. a classification prediction or a regression score; and final decisions are the decisions made by the clinician that may be informed by the algorithmic decision, e.g. a diagnosis or treatment recommendation. ‘Algorithmic decisions’ and ‘final decisions’ can be distinguished by their causal role. When the algorithm provides a human decision-maker with a probability score,

then this score is causally upstream to the final decision, whereas the final decision is causally relevant (either as the diagnostic outcome or by culminating in a given treatment choice).

Given such a view, the appropriate locus of evaluation for fairness judgements is final decisions. The patient has a fairness complaint only if and because, and to the extent that, the final decision is unfair. Here the sense in which a final decision may be unfair is what Hedden (2021) calls fairness in virtue of group membership. That is: The sort of fairness complaint that a patient may have with respect to final decisions is, for example, that diagnoses for a group characterised by a protected characteristic to which the patient belongs are less accurate or have a higher false negative rate than other groups due to bias arising at some point in the decision-making process. The bias could be located in the algorithmic decision, but it could also consist in the healthcare professional’s failure to identify and correct for algorithmic bias.

However, what is important to understand is ‘fair algorithmic decision-making’ relative to certain statistical fairness criteria does not imply fair final decision-making, where fair final decision-making is straightforwardly the relevant sense of fairness insofar as unfair final decisions are what ultimately impact patient wellbeing. It might be the case that independent biases exist in the human part of the decision-making process, such that the satisfaction of relevant statistical criteria for fairness does not guarantee a fair final decision. For example, a study by Green and Chen (2019) investigated how the use of predictive models affects actual decision-making processes within the context of criminal justice. By way of an experiment on Mechanical Turk, the study found that if provided with a risk assessment, human decision-makers often deviate from algorithmic risk assessments. Especially for Black defendants, the use of algorithmic risk assessments led to higher risk scores, while for white defendants, the involvement of human decision-makers led to a decrease in the risk score. Green and Chen refer to this as *disparate interaction*. We are not aware of a likeminded study within the context of healthcare. However, it is certainly not implausible to assume that ‘disparate interaction’ can also affect clinical decisions, such as the grading of knee pain. Furthermore, a study from Tschandl et al., (2020) develops a nuanced picture in how particularly novice clinicians tend to be over-reliant on algorithmic support, whereas expert clinicians are more likely to stick to their own diagnoses.

Moreover, ‘unfair algorithmic decision-making’ relative to certain statistical criteria does not imply unfair final decision-making. Biases exhibited by algorithms can be identified and corrected for in the human part of the decision-making process. Of course, this is easier said than done, in light of the opacity of many ML algorithms (cf. Creel, 2020), as well as the intrinsic uncertainty and time constraints involved in clinical decision-making.

Accordingly, while statistical fairness criteria make biases in ML algorithms quantifiable and tangible as part of the model evaluation process (Hardt & Recht, 2021: 38), and whilst it might be warranted to assume some association between statistical criteria for ‘fair algorithmic decision-making’ and fair ‘final decisions’ in that the involvement of biased algorithmic decisions will oftentimes result into unfair final decisions, the link is not a causal one. Finally, even when emphasizing final decisions, statistical fairness criteria remain meaningful for measuring fairness.

## Collaboration Between Clinicians and ML Models

In this section and the next, we articulate and defend an account of the conditions under which ML algorithms in healthcare may permissibly overfit for socially and racially disadvantaged groups. The aim of this section is, first, to make precise three models of collaboration between clinicians and ML algorithms, i.e. accounts of how and in what respects clinician judgement and ML regression and classification predictions can together produce a final decision such as a diagnosis or treatment recommendation. Second, once the plausible models of collaboration are clear, we develop a normative ideal for clinician-algorithm collaboration. According to what we call the ‘division of labour standard’, the normative ideal for collaboration between clinicians and algorithms is that the distribution of clinical tasks to clinicians and algorithms is efficient. What this means is that the allocation of clinical tasks in question is not worse than any other relative to the health outcomes produced, where health outcomes are evaluated holistically so as to include aggregate patient welfare, alongside patient satisfaction and fairness across groups.

### The Peer Model

The simplest model of clinician-algorithm collaboration is what we can call the ‘peer model’. According to the peer model, ML algorithms and clinicians offer competing predictions about, for example, the correct diagnosis or the best treatment recommendation. The salient feature of this account is that clinicians and algorithms address the same clinical task, and their solutions to that task then need to be balanced.

In practice, the peer model may be instantiated in one of two respects. On the one hand, it may be that the clinician and ML algorithm each offer, say, a diagnosis, taking into account relevant clinical evidence about the patient’s condition; and then the two diagnostic predictions are aggregated to produce an overall diagnosis. How exactly this aggregation procedure works is an open question. But one plausible suggestion is that the clinician’s subjective probability distribution over candidate diagnostic hypotheses is summed with the algorithm’s probability distribution, and then normalised to produce an aggregate distribution. Then, on this view, the aggregate distribution is used to inform clinical decisions about which diagnostic hypotheses are pursued and in what order when determining a final diagnosis.

On the other hand, it may be that the ML algorithm functions as a second medical opinion. Here the idea is that the clinician gives, say, a treatment recommendation, and the algorithm also provides a treatment recommendation. Then the clinician may take into account the algorithmic treatment recommendation in much the same way that they would treat a second medical opinion. That is, if the ML algorithm agrees with the clinician about the optimal treatment plan, then the clinician may proceed with confidence with that recommended treatment. But if the two disagree, then the clinician must engage in a justificatory process to determine the considerations in favour of and against both treatment recommendations, and ultimately recommend to the patient the treatment for which the clinical case is strongest (Kempt & Nagel, 2021). The use of ML algorithms as a second opinion has been explored in a study by McKinney et al., (2020), showing that the involvement of the algorithm improves the overall accuracy, while decreasing the clinicians’ workload in mammography screenings.

The peer model, if true, provides a plausible rationale for a method of model validation that has featured in several prominent papers about ML in healthcare. In particular, studies that use an antagonistic framing of algorithms competing versus clinical experts, with the former being at least as good or strictly better than the latter, presume that the role of ML algorithms is akin to that of a peer (Gulshan et al., 2016; Esteva et al., 2017; De Fauw et al., 2018; see also Bjerring and Busch, 2021 ; Grote & Berens 2020; Grote & Berens, 2021). While studies indicate, that the combination of a clinician and a ML algorithm bring about more accurate diagnoses as opposed to the clinician deciding in isolation (McKinney et al., 2020; Tschandl et al., 2020), the peer model is to some degree unattractive as it has been shown that clinicians are prone to over-rely on algorithmic advice, thus being led astray when the algorithm decides incorrectly (Tschandl et al., 2020; Gaube et al., 2021). Furthermore, despite providing epistemic benefits, the peer model gives rise to concerns that have to do with informed consent and patient-centred care (cf. Keeling & Nyrup (forthcoming)). In particular, McDougall (2019: 157) argues that treatment recommender systems render the clinical decision-making process such that “individual patient’s values do not drive the ranking of treatment options.” The worry is that algorithms that recommend treatments will in some sense replace the clinician’s advisory role, and do so in such a way that traditional ethical norms such as informed consent and patient-centred care are difficult or impossible to satisfy. What is concerning is that ML algorithms used in healthcare somehow threaten clinicians as epistemic authorities in clinical decisions, and that despite possible gains in accuracy, other critical functions of clinicians will be overlooked.

### The Triage Model

While the ‘peer model’ exemplifies the threats to the epistemic authority of clinicians through implementing ML algorithms in clinical environments, it is not the only way in which the relevant collaboration can manifest. Indeed, a growing strand of research explores how ML algorithms and clinicians can complement each other (Raghu et al., 2019).^9^ This section considers what we can call the ‘triage model’. The idea is that an algorithm’s prediction may be causally upstream of the clinician’s judgement such that the clinician’s judgement is enhanced in virtue of the clinician’s knowledge of the prediction.

Consider an example from ophthalmology. First, some applications of ML in medicine are intended to mitigate the impact of shortages of specialised clinicians in the developing world. For example, Google Health developed a ML algorithm for detecting early onset diabetic retinopathy based on retina scans (Gulshan et al., 2016; see also Poplin et al., 2018). The upshot of this collaborative model is that clinical tasks typically reserved for expert clinicians can be delegated to ML algorithms in circumstances where the prevalence of a disease exceeds the capacity of clinicians to detect the disease given specialist clinician shortages. Once early onset of the relevant condition – be it diabetic retinopathy or something else, has been detected by the algorithm, the patient can then be referred to a specialist. Accordingly, the role of the ML algorithm is to predict whether to defer a diagnostic task to an expert clinician.

The benefit of this model of clinician-algorithm collaboration is that the algorithm alleviates the burden on clinicians given that expert clinicians in the relevant domain are scarce. The upshot is that patients who require clinical judgement may be referred to the appropriate expert clinicians in advance of their condition deteriorating and at relatively low cost to healthcare providers. Here the cost is low because no human clinicians are required to perform the initial pre-diagnostic risk assessment. However, a potential downside of such algorithmic pre-screenings is that the might drive confirmation bias in clinicians, making an independent assessment of patients challenging. Another downside regards positive cases, not spotted by the algorithm (false negative) are not referred to specialists. Thus, even if the algorithm has a high predictive performance in general, a fraction of cases will be penalized.

### The Auditing Model

The ‘triage model’ is such that the algorithm’s role is to make a judgement *upstream* of the clinician’s judgement. The ‘auditing model’, in contrast, is such that the algorithm’s judgement is *downstream* of the clinician’s judgement. Roughly, the role of ML systems on this model is to act in a supervisory or regulatory capacity. The auditing model could be instantiated in at least two different respects. First, the ML model could supervise the clinician’s performance at a particular task such as diagnosis or treatment recommendation. One straightforward example of this setup is where the role of the clinician is to reach a diagnosis based on their assessment of the patient’s case. The clinician’s diagnosis is then checked against a differential diagnosis provided by the ML algorithm, i.e. a list of plausible diagnostic hypotheses given the available evidence, and then a red flag is raised only if the clinician’s diagnosis is not included in the algorithm’s differential diagnosis. The effect, then, is run a plausibility check on the clinician’s judgement to ensure that the diagnosis offered is at least plausible. This supervisory setup may be most beneficial for medical students and junior clinicians, and may alleviate the supervisory burden on senior clinicians to ensure quality standards in diagnosis for more junior clinicians. Indeed, ML auditing systems could be employed to flag situations in which a second opinion from a senior colleague is advisable. The auditing model of clinician-algorithm collaboration is likely to be widely applicable in healthcare settings given the prevalence of on-the-job training for novice clinicians.

Second, the ML algorithm could supervise the clinician’s performance across several tasks in an appropriate comparison class. For example, a cardiologist’s performance at recognising different kinds of cardiac conditions based on electrocardiogram (ECG) data. Here the algorithm’s role is that of an auditor. In particular, the algorithm may be utilised for the detection of biases across different sub-populations of patients. What is suggested being here is that the clinician may exhibit differential success in predicting the presence or absence of different cardiac conditions in light of certain features of patients. For example, patients who are regular users of stimulants such as cocaine may exhibit irregular cardiac behaviour such that the detection of cardiac conditions is more difficult in these patients than in non-cocaine-using patients. Informing a cardiologist that they systematically underperform on diagnostic tasks for patients who are regular cocaine users is a useful piece of information for the clinician, at least insofar as it provides a plausible locus of concentration for improving the clinician’s diagnostic ability. Hence use of ML algorithms to identify which sub-populations of patients are such that the clinician systematically underperforms on those sub-populations could greatly improve health outcomes.

 Finally, the ML algorithm could be used to detect markers of sub-optimal performance in clinicians, for example, fatigue. To illustrate: Baghdadi et al., (2018) developed a proof-of-principle ML algorithm to predict whether or not individuals are fatigued following a demanding manual occupational task such as heavy lifting (see also Baghdadi et al., 2021; Hernandez et al., 2020). The algorithm relied on sensor readings taken from a non-invasive ankle bracelet, and displayed 90% accuracy on the validation set. Because clinical medicine involves routine physical demands, such as moving patients and rapid response to emergencies, the onset of fatigue could reduce clinical performance across a range of physical and cognitive tasks. Hence, the use of ML systems for fatigue monitoring and detection offers a plausible means to reduce morbidity and mortality arising from clinical errors that result from clinician fatigue. For example, the algorithm could alert clinicians to seek a second opinion on clinical judgements made when fatigued. A caveat, however, is that there is a fine line between a auditing system providing decision-support and paternalistic interference – thereby threatening the clinician`s epistemic authority.

### Division of Labour

We have examined three models of the collaborative relationship between clinicians and ML algorithms: the ‘peer model’, the ‘triage model’, and the ‘auditing model’. To determine the conditions under which each model of clinician-algorithm collaboration is appropriate, it is necessary to offer a normative standard against which clinician-algorithm collaboration can be evaluated. Here we propose the ‘division of labour model’. What motivates the division of labour is the observation that there is an opportunity cost to clinicians performing any particular clinical task. For example, the fact that a clinician spends some amount of time assessing whether or not a patient in the emergency room is likely to require specialist observation and treatment due to sepsis implies that the clinician does not spend that same amount of time performing other clinical tasks. Thus, using an algorithm to detect sepsis frees-up the time that the clinician would otherwise spend conducting a quick sepsis-related organ-failure assessment (qSOFA) (Kim et al., 2020: 163; Moor et al., 2021).

Because certain tasks can *only* be performed by clinicians, such as ensuring that the patient has sufficient understanding of their situation to provide informed consent to an intervention, the rationale behind the division of labour model is that clinician time is best allocated to tasks that can only be performed by clinicians, or are best performed by clinicians. What matters, on the view being defended, is that clinical labour is divided between clinicians and algorithms so as to ensure that clinician time is used optimally with respect to health outcomes and patient satisfaction. Accordingly, the division of labour model emphasises *optimal collaboration* and not *competition* between clinicians and algorithms. Intriguingly, facilitating optimal collaboration might involve that the respective strengths of clinicians and algorithms in some task lie in different areas (Wilder et al., 2020; Bansal et al., 2021).^10^

Along these lines, Keeling and Nyrup (forthcoming)  have characterised the relation between ML algorithms and clinicians as one of ‘cognitive offloading’. The idea is that the clinician can offload certain cognitive tasks onto the algorithm in order to focus on other tasks. Provided clinicians and ML algorithms are assigned tasks efficiently, i.e. there is no alternative task allocation that will result in better health outcomes, then such cognitive offloading is desirable. Here we propose to evaluate health outcomes holistically. What this means is that the evaluation of health outcomes is not reducible to a simple metric such as maximising quality-adjusted life-years (QALYs) per dollar spent, but rather the evaluation includes broader considerations such as patient trust and satisfaction, and also the fairness of health outcomes across protected groups. The proposal, then, is to distribute tasks between clinicians and algorithms in a way that is best suited to promoting a range of goods including patient health, patient satisfaction, and fairness. To be sure, this normative standard is compatible with use of the ‘peer model’, ‘triage model’ and the ‘auditing model’, provided each model of clinician-algorithm collaboration is deployed in the right circumstances, i.e. the setup reflects the best division of tasks between clinicians and algorithms relative to promoting good health outcomes broadly construed. As will be discussed in the next section, successfully implementing division of labour solutions entails various morally-relevant caveats.

To summarise: The upshot of this section is that there are at least three plausible models for clinician-algorithm collaboration, and a plausible normative ideal to aim at in deciding on how to allocate tasks between clinicians and algorithms is the division of labour standard. According to this standard, what matters is that clinical tasks are allocated efficiently between clinicians and algorithms, so as to minimise the opportunity cost on clinicians performing any particular task. Because the division of labour standard operates with a holistic conception of the evaluation of health outcomes, taking into account patient welfare alongside patient satisfaction and fairness, the division of labour model offers a plausible normative backdrop against which to consider the conditions under which an algorithm may permissibly overfit for socially disadvantaged groups in order to ensure fair final decisions. In the next section, we characterise the sorts of unfairness that can be tolerated in ML decisions under different models of algorithm-clinician for collaboration, and in doing so, make the case for the permissibility of affirmative algorithms in certain circumstances.

## From Biased Algorithms to Fair Final Decisions

In order to showcase how decisions from a biased algorithm can culminate into fair final decisions, we need to make some background assumptions. First, we consider a clinical setting in which the algorithm developed by Pierson et al. (2021) is used to support a clinician in the diagnosis of pain. Second, we focus on a binary classification task, in which the accuracy of final decisions for Black and white patients is being measured. Moreover, the clinician and the ML algorithm are roughly equal in terms of diagnostic accuracy. As a fairness metric, any classification parity notion will do.

Here, the clinician`s accuracy for Black patients is 0.7 and 0.9 for white patients, whereas for the algorithm it is the other way around. We acknowledge that this highly idealized framing comes at some costs – especially since it is unable to capture issues of fairness in intersectional categories. However, this problem is hardly unique to our approach and haunts the general debate on statistical fairness criteria in ML. Finally, we assume that the clinician and the algorithm use the same evidence for their diagnosis (X-ray images). Again, this might be deemed as problematic insofar self-reports (“how bad does it hurt?”) and other diagnostic modalities are being ignored.

With that in mind, consider how the interplay of the clinician and the ML algorithm can bring about fair final decisions. Let us start with the ‘peer model’. Here, the logic is simple. If the clinician is inclined to defer (most) decisions to the algorithm in virtue of peer pressure, the final decisions will in all likelihood be more accurate for Black patients than for white patients. Thus, when measured in the prevalent statistical fairness criteria, the final decisions will be unfair. The situation improves if the clinician and the algorithm aggregate their judgments – which in this case might involve taking the mean value of their individual pain severity scores. Since the clinician and the algorithm aggregate their pain severity scores, the outcome of the final decisions will be fair across different sub-populations (with an overall accuracy of 0.8). However, despite being fair, the overall accuracy tends to be lower than other approaches to be discussed in this section.

Let us move on to the ‘division of labour model’. We might start by considering a crude variant of this model, in which the algorithm is used to diagnose Black patients, whereas the clinician is supposed to diagnose pain for white patients. Since for both groups of patients, the diagnostic accuracy would roughly be 0.9, the relevant division of labour results into final fair decisions. That said, despite enabling fairness, the crude division of labour model has some unbearable ethical implications. For a start, delegating diagnostic tasks of underserved groups to an algorithm, whereas their more privileged counterparts are assigned to a human clinician might be deemed as degrading – as it destined to be detrimental to the clinician-patient-relationship. Another potential pitfall in this solution is that it might preserve biases in the clinician, as there is no incentive for her to critically reflect on her implicit biases and prejudices, as well identifying appropriate means to overcome them.^11^ Consequently, while the crude division of labour model might improve the fairness of diagnostic decisions, it might perpetuate or even reinforce health inequities across other dimensions.

Now, take a less crude version of the ‘division of labour’ model, in which the clinician and the algorithm initially evaluate the pain severity in the X-ray image independently, while also estimating their confidence in their judgment. In cases, in which the confidence of the clinician falls below a pre-defined threshold and in which the algorithm has high confidence, the clinician defers to the algorithm.^12^ Assuming that their confidence scores are well-calibrated, the clinician will in all likelihood be more confident when inspecting X-rays from white patients, whereas the algorithm will be more confident with Black patients. Similar to the crude ‘division of labour model’, the accuracy for Black and white patients will be 0.9. Moreover, given that there is more involvement from the clinician, the solution proposed might be less detrimental to the clinician-patient-relationship. However, the revised version of the division of labour model also has some downsides, in that it is cognitively costly (the clinician and the algorithm both have to make individual diagnoses) and the problem of *bias preservation* in clinicians is left unaddressed.

A way to accommodate bias preservation might be by assigning a hybrid role to the algorithm, in which it acts both as a peer and an instructor. In this vein, Pierson et al. recommend supplementing their algorithm with a heatmap (2021: 139).^13^ In highlighting regions of interest in the algorithm`s diagnosis, a clinician might learn which areas to direct her attention to. Thus, the explanations provided by the heatmap have a primarily pedagogical function. Plausibly, after a period of interacting with the algorithm, the clinician might adapt her diagnostic standards, when examining patients from a given sub-population. However, to ensure that the instructions made by the algorithm do indeed translate into more accurate pain scores for Black patients, additional safeguards need to be implemented. In particular, clinicians might be required to diagnose a fraction of Black patients themselves (even if they are less confident than the algorithm), while receiving further supervision – be it from a fellow colleague or another algorithm. Moreover, to ensure that the interplay of ML algorithms and clinicians indeed is beneficial to patients, it necessary to also control for patient outcome measures – e.g., QUALYs .

## The Threat of Solutionism

We have defended the permissible use of biased ML algorithms provided the models further the aim of health equity. However, our analysis has been confined to a narrow clinical setting. Since health injustices typically result from a concatenation of social, economic, and psychological factors, it might be objected that our proposed solution fails to register the moral complexity of what is at issue. Indeed, there is a risk of running into a ‘solutionist’ trap in trying to fix social problems through technological interventions – which might even backfire and exacerbate health injustices (cf. Morozov, 2013 for the concept of ‘solutionism’). This concern merits serious consideration since existing attempts to rectify injustices via ML algorithms do not have an impressive track record (Noble, 2018). At the same time, it would be remiss to ignore the opportunities that ML algorithms provide to remedy health disparities, given that relevant issues have haunted healthcare for decades. Against this backdrop, we set out to lay the grounds for an auditing of ML algorithms in healthcare, considering fairness as a system-level property (Holstein et al., 2019). For this purpose, we identify a set of necessary conditions for the permissible deployment of affirmative algorithms in healthcare, acting as safeguards against the improper use of ML algorithms for solving complex social problems. The algorithm developed by Pierson et al., (2021) will again be used as an example to underscore our claims. We take it that our approach shares the spirit of other holistic evaluation guidelines for ML algorithms within the context of healthcare (see Zicari et al., 2021).

**Purposiveness**: A first issue is whether a given algorithmic intervention is an adequate means for a given purpose. This makes it necessary to specify its intended function: the rationale for its use and how it is supposed to be used by clinicians. Regarding the algorithm from Pierson et al., matters arise due to the absence of a causal hypothesis explaining why pain differences in KLG between Black and white people can be attributed to knee-internal factors. If pain differences turn out to be caused by factors external to the knee, such as stress or physically more demanding jobs, then the increase in surgeries will do more harm as the symptoms are likely to re-occur. Similarly, instead of an algorithm, a simpler solution might be to develop a diagnostic standard, generalizing better across different sub-populations. In sum, to determine whether an algorithmic intervention is adequate for its intended purpose, we need a functional specification in addition to evidential support for is efficacy.

### Functional Stability

Many algorithmic interventions undergo function shifts over time (cf. Koops, 2021). For instance, while an algorithm might initially be used to improve the performance of teachers, the very same algorithm could later be used for the surveillance of teachers (cf. O`Neil, 2016, pp. 4–5). Likewise, an algorithm designed to remedy health injustices might reinforce health disparities once there is a change in its function. Applied to our running example, a clinic might feel inclined to move from a less crude, towards a crude variant of ‘division of labour’ for economic reasons. The problem with such function shifts is that they typically happen gradually and subtly. Hence, the detection of function shifts is difficult, and the only plausible safeguards are to be transparent about the intended function of an algorithm and having regular auditing.

### Holistic evaluation

Successfully overcoming health-disparities across different sub-populations requires a concentrated effort. While an algorithmic intervention might improve the outcome regarding one metric, it may fare worse in respect to others. In the case study on the algorithm developed by Pierson et al., one such trade-off might relate to an increase in diagnostic accuracy for socially disadvantaged groups, at the expense of the clinician-patient relationship. Consequently, any algorithmic intervention aimed at mitigating health disparities should be wary of relying on reductionist evaluation criteria. Similarly, algorithmic interventions might backfire if other means to remedy health-disparities are cut in return – e.g., interventions aimed at reducing (implicit) stereotypes among medical professionals (see Owens & Walker 2020 for an overview on anti-racist practices in medical research and practice).

### Anticipation and participation

Since any algorithmic intervention may have wide-ranging consequences that go beyond its intended effects, it is crucial to anticipate potential risks. Here, a representation from diverse perspectives is particularly important – especially from those who might be affected the most from the planned algorithmic intervention. A possible framework to bring together the points of view of different stakeholders might be ‘value-sensitive design’ (Friedman and Hendry, 2019). An inclusion of diverse viewpoints is also necessary to facilitate co-determination rights for vulnerable social groups within the healthcare system.

### Robust evidence

The arguably most critical issue before implementing an algorithmic intervention for the purpose of mitigating health disparities relates to whether there is any good evidence that it works. The evaluation of ML algorithms is typically confined to their statistical properties. However, this is uninformative with regard to its performance within a socio-technological system, such as clinical environments. Much to our surprise, there is no mentioning in the proposal for the regulation of AI/ML systems by the European Commission (2021) of how externally valid evidence should be established that a safety-critical algorithmic intervention is indeed reliable/beneficial. In contrast, we think that it is indispensable to raise such methodological issues. Most importantly, it needs to be identified when and how field trials – let alone randomized controlled trials (RCTs) – should be conducted for algorithmic interventions.^14^ That said, as RCTs may have their shortcomings when applied to ML algorithms, we suggest combining them with ethnographic or other types of qualitative studies (cf. Genin & Grote, 2021).

While the set of criteria is far from being exhaustive, we hope that it underlines the importance of a holistic approach in the evaluation of algorithmic interventions aimed at mitigating social problems.

## Conclusion

In this paper, we defended the permissible deployment of affirmative ML algorithms in clinical medicine that overfit for minority and socioeconomically disadvantaged sub-populations, and thus perform better on the relevant sub-populations than for traditionally advantaged sub-populations. In particular, we argued that such algorithms can permissibly be deployed provided they serve the aim of health equity. Affirmative algorithms can serve the aim of health equity, we argued, on certain models of collaboration between clinicians and ML algorithms. Because the fairness of *final decisions*, which result from collaboration between clinicians and algorithms, is the appropriate locus of moral concern, unfair *algorithmic decisions* can under the right conditions be tolerated. Namely, if, and only if, the resultant final decisions are fair. Finally, we registered, and attempted to mitigate, the concern that our proposal is an instance of ‘solutionism,’ i.e. the practice of attempting to use quick-fix algorithmic interventions that fail to account for the socioeconomic complexity of the injustices at issue. We offered a series of necessary conditions on the permissible use of affirmative algorithms in order to combat or at least mitigate the threat of solutionism.

We shall conclude with three main takeaways. First, the mechanisms through which ML can exacerbate and amplify existing social injustices are at this point widely understood. What is less explored is how and in what respects ML can address and mitigate existing injustices. Healthcare is one domain in which careful reflection on structural injustices, in addition to the imaginative development and application of novel ML technologies, could in principle resolve, or at least mitigate, certain injustices against minorities. Second, at least in the domain of healthcare, ML technologies are principally deployed as decision support systems. Hence overemphasis on the evaluative properties of algorithmic decisions, as opposed to the evaluative properties of the collaborative process that facilitates joint clinician-ML decision-making, is likely to hinder efforts to rectify injustices. What matters, instead, is that the processes by which ML algorithms and clinicians *jointly* contribute to final decisions are sufficiently well understood to determine exactly how algorithms can be designed and deployed in a way that promotes equity in health outcomes. Third, the complexity of the nature and causes of social injustices in healthcare should not be underestimated. There are no ‘quick fix’ algorithmic solutions. However, with a participatory approach to developing affirmative ML systems that takes as central the views and interests of minority groups, there are plausible grounds for supposing that ML technologies can be levied to combat and mitigate social injustices in healthcare, provided appropriate safeguards are put in place.

## Footnotes

1 Here underperformance for a particular sub-population can be characterised in terms of, *inter alia*, disparities in false positive rates, false negative rates, and overall accuracy, for that sub-population relative to others (Kleinberg et al., 2016; Chouldechova, 2017; Miconi, 2017). See Sect. 3 for discussion.

2 Note, however, that the study does not consider the clinical applicability of the algorithm. Rather, it might be deemed as a ‘proof of concept’ study, exploring the use of ML algorithms to mitigate health disparities. We thank an anonymous reviewer for raising this point.

3 See also Roos & Lohmander (2003).

4 Note that there are also possible downsides to the increase in accuracy, in that it might cause overtreatment. We discuss this also in Sect. 6. We thank an anonymous referee for this comment.

5 Note that ‘bias’ is a multi-faceted concept, with different senses and sources. For a detailed discussion of the mechanisms and moral evaluation of biases in ML, see Johnson 2020; Fazelpour & Danks, 2021; authors, b.

6 Importantly, the researchers did not compare the algorithm`s predictive performance between white and Black patients.

7 For a comprehensive overview on statistical fairness criteria, see Barocas et al., 2019, ch. 2–3. See also Mitchell et al., (2021)

8 For a discussion on statistical fairness criteria and broader claims of justice, see Zimmermann & Lee-Stronach 2021).

9 Indeed, empirical research on decision support systems in clinical practice has found that low user acceptance among clinicians is in large part explained by poor integration as opposed to poor algorithmic performance (Jacobs et al., 2021; see also Khairat et al., 2018).

10 Importantly, research on ‘division of labour’ is currently still concerned with algorithmic developments and has not been clinically validated.

11 See Holroyd 2012, 2017 for a useful overview on ethical issues regarding implicit biases and prejudices.

12 For a discussion of uncertainty quantification in ML algorithms, see Kompa et al., 2021.

13 .

14 See Deaton & Cartwright 2018; Cartwright & Hardie, 2012 for a pronounced treatment of methodological issues in RCTs.

## Legal Documents

European Commission: Proposal for a REGULATION OF THE EUROPEAN PARLIAMENT AND OF THE COUNCIL LAYING DOWN HARMONISED RULES ON ARTIFICIAL INTELLIGENCE (ARTIFICIAL INTELLIGENCE ACT) AND AMENDING CERTAIN UNION LEGISLATIVE ACTS.
